# Efficacy and Safety of Hydroxychloroquine vs Placebo for Pre-exposure SARS-CoV-2 Prophylaxis Among Health Care Workers: A Randomized Clinical Trial

**DOI:** 10.1001/jamainternmed.2020.6319

**Published:** 2020-09-30

**Authors:** Benjamin S. Abella, Eliana L. Jolkovsky, Barbara T. Biney, Julie E. Uspal, Matthew C. Hyman, Ian Frank, Scott E. Hensley, Saar Gill, Dan T. Vogl, Ivan Maillard, Daria V. Babushok, Alexander C. Huang, Sunita D. Nasta, Jennifer C. Walsh, E. Paul Wiletyo, Phyllis A. Gimotty, Michael C. Milone, Ravi K. Amaravadi, and the Prevention and Treatment of COVID-19 With Hydroxychloroquine (PATCH) Investigators

**Affiliations:** 1 Department of Emergency Medicine, University of Pennsylvania, Philadelphia; 2 Division of Cardiology, Department of Medicine University of Pennsylvania, Philadelphia; 3 Division of Infectious Disease, Department of Medicine University of Pennsylvania, Philadelphia; 4 Department of Microbiology, University of Pennsylvania, Philadelphia; 5 Abramson Cancer Center and Division of Hematology-Oncology, Department of Medicine, University of Pennsylvania, Philadelphia; 6 Department of Biostatistics, Epidemiology and Informatics, University of Pennsylvania, Philadelphia; 7 Department of Pathology, University of Pennsylvania, Philadelphia

## Abstract

**Importance:**

Health care workers (HCWs) caring for patients with coronavirus disease 2019 (COVID-19) are at risk of exposure to severe acute respiratory syndrome coronavirus 2 (SARS-CoV-2). Currently, to our knowledge, there is no effective pharmacologic prophylaxis for individuals at risk.

**Objective:**

To evaluate the efficacy of hydroxychloroquine to prevent transmission of SARS-CoV-2 in hospital-based HCWs with exposure to patients with COVID-19 using a pre-exposure prophylaxis strategy.

**Design, Setting, and Participants:**

This randomized, double-blind, placebo-controlled clinical trial (the Prevention and Treatment of COVID-19 With Hydroxychloroquine Study) was conducted at 2 tertiary urban hospitals, with enrollment from April 9, 2020, to July 14, 2020; follow-up ended August 4, 2020. The trial randomized 132 full-time, hospital-based HCWs (physicians, nurses, certified nursing assistants, emergency technicians, and respiratory therapists), of whom 125 were initially asymptomatic and had negative results for SARS-CoV-2 by nasopharyngeal swab. The trial was terminated early for futility before reaching a planned enrollment of 200 participants.

**Interventions:**

Hydroxychloroquine, 600 mg, daily, or size-matched placebo taken orally for 8 weeks.

**Main Outcomes and Measures:**

The primary outcome was the incidence of SARS-CoV-2 infection as determined by a nasopharyngeal swab during the 8 weeks of treatment. Secondary outcomes included adverse effects, treatment discontinuation, presence of SARS-CoV-2 antibodies, frequency of QTc prolongation, and clinical outcomes for SARS-CoV-2–positive participants.

**Results:**

Of the 132 randomized participants (median age, 33 years [range, 20-66 years]; 91 women [69%]), 125 (94.7%) were evaluable for the primary outcome. There was no significant difference in infection rates in participants randomized to receive hydroxychloroquine compared with placebo (4 of 64 [6.3%] vs 4 of 61 [6.6%]; *P* > .99). Mild adverse events were more common in participants taking hydroxychloroquine compared with placebo (45% vs 26%; *P* = .04); rates of treatment discontinuation were similar in both arms (19% vs 16%; *P* = .81). The median change in QTc (baseline to 4-week evaluation) did not differ between arms (hydroxychloroquine: 4 milliseconds; 95% CI, −9 to 17; vs placebo: 3 milliseconds; 95% CI, −5 to 11; *P* = .98). Of the 8 participants with positive results for SARS-CoV-2 (6.4%), 6 developed viral symptoms; none required hospitalization, and all clinically recovered.

**Conclusions and Relevance:**

In this randomized clinical trial, although limited by early termination, there was no clinical benefit of hydroxychloroquine administered daily for 8 weeks as pre-exposure prophylaxis in hospital-based HCWs exposed to patients with COVID-19.

**Trial Registration:**

ClinicalTrials.gov Identifier: NCT04329923

## Key Points

**Question:**

Does a regimen of hydroxychloroquine, 600 mg, per day, reduce the transmission of severe acute respiratory syndrome coronavirus 2 (SARS-CoV-2) as a pre-exposure prophylaxis strategy when taken by hospital-based health care workers?

**Finding:**

In this double-blind, placebo-controlled randomized clinical trial that included 132 participants and was terminated early, there was not a significant difference in reverse-transcriptase polymerase chain reaction–confirmed SARS-CoV-2 incidence between hydroxychloroquine and placebo cohorts.

**Meaning:**

Among hospital-based health care workers, daily hydroxychloroquine did not prevent SARS-CoV-2 infection, although the trial was terminated early and may have been underpowered to detect a clinically important difference.

## Teaser

This randomized clinical trial evaluates the efficacy of hydroxychloroquine to prevent transmission of SARS-CoV-2 in health care workers with exposure to patients with COVID-19 at 2 hospitals in Philadelphia, Pennsylvania.

## Introduction

The pandemic triggered by severe acute respiratory syndrome coronavirus 2 (SARS-CoV-2) has affected more than 6.8 million people in the US, with more than 200 000 deaths to date.^1,2^ The illness caused by the SARS-CoV-2 virus, coronavirus disease 2019 (COVID-19), has spread broadly with significant effects on elderly and minority individuals, those with significant comorbidities, and members of the health care workforce.^3,4,5,6^ Public health measures to prevent COVID-19 disease have largely depended on physical distancing, use of facial covers and personal protective equipment (PPE), and hand hygiene.^7^ Health care workers (HCWs) assigned to treating patients with COVID-19 have frequent potential exposures, raising the question of whether pharmacologic prophylaxis is warranted.

Medication-based prevention and treatment of COVID-19 have proven challenging. To our knowledge, to date, only 2 medications, dexamethasone and remdesivir, have been shown to improve outcomes in severe COVID-19 disease,^8,9^ and no treatment has proven effective in mild to moderate disease. Furthermore, no pharmacologic prophylaxis for COVID-19 has been established. Given the many HCWs with substantial COVID-19 exposure worldwide, there is great interest in finding an effective medication to prevent viral transmission.

Hydroxychloroquine, a 4-aminoquinoline with antimalarial and antiautophagic properties, has been identified as a possible prophylactic medication for SARS-CoV-2.^10,11^ Hydroxychloroquine has been widely used since its US Food and Drug Administration approval in 1956 for treating systemic lupus erythematosus and is generally well tolerated, with few long-term adverse effects.^12,13^ A recent randomized trial for postexposure COVID-19 prophylaxis with a 5-day course of hydroxychloroquine did not demonstrate clinical benefit.^14^ However, the composite primary outcome measure for this study included symptoms consistent with infection without laboratory confirmation; most patients did not have assessment of SARS-CoV-2 infection by reverse-transcriptase polymerase chain reaction (RT-PCR), raising concerns of type II error from asymptomatic participants.^15^ We sought to test the hypothesis that administering daily hydroxychloroquine would prevent SARS-CoV-2 infection in hospital-based HCWs over 8 weeks of exposure via RT-PCR testing of nasopharyngeal (NP) swabs and serologic antibody testing from participants at baseline, 4 weeks, and 8 weeks of treatment.

## Methods

This single-health system, double-blind placebo-controlled randomized trial was conducted as the prophylaxis substudy of the Prevention and Treatment of COVID-19 With Hydroxychloroquine (PATCH) investigations at 2 hospitals within the Penn Medicine system: the Hospital of the University of Pennsylvania, a 839-bed teaching hospital, and Penn Presbyterian Medical Center, a 375-bed teaching hospital (Philadelphia, Pennsylvania). Participation spanned from April 9, 2020, to July 14, 2020, during which both hospitals had uniform policies for HCW use of PPE (including masks, eyewear, and gowns) as well as patient screening for COVID-19 symptoms. An independent medical monitor, data safety monitoring board (DSMB), and COVID-19 trial steering committee provided oversight of safety and efficacy end points. Electronically signed informed consent was obtained from all participants via direct communication with a member of the physician investigative team. Consent was conducted via an internet document signature program (DocuSign; DocuSign Inc) that was compliant with US Food and Drug Administration 21 CFR Part 11 regulations for signature verification. Approval for the study was granted by the University of Pennsylvania institutional review board. The study protocol and statistical analysis plan are included as Supplement 1 and Supplement 2, respectively. The protocol and manuscript were prepared following the Consolidated Standards of Reporting Trials guidelines for randomized clinical trials.

### Study Participants

Health care workers at either study hospital were eligible for inclusion if they (1) worked 20 hours or more per week in hospital-based units, (2) had no known history of SARS-CoV-2 infection, and (3) did not have symptoms suggestive of COVID-19 in the 2 weeks before enrollment, including cough, fever, or shortness of breath. Physicians, nurses, certified nursing assistants, emergency technicians, and respiratory therapists were eligible. Enrollment was focused on staff members in the emergency department and dedicated COVID-19 units. Exclusion criteria included history of (1) a positive SARS-CoV-2 test result, (2) allergy or sensitivity to hydroxychloroquine, (3) glucose-6-phosphate dehydrogenase deficiency, (4) retinal diseases, such as macular degeneration or diabetic retinopathy, and (5) substantial cardiac disease (eg, arrhythmia, congestive heart failure, or coronary disease); other exclusion criteria are listed in Supplement 1. Demographic information was obtained from participants directly, including self-report of race/ethnicity.

### Safeguards Against Coercion

Recruitment efforts were made by study investigators who were not in any direct supervisory role or in the same department as the potential HCW study participant. The consenting investigator reminded potential enrollees that the decision to participate would not affect performance evaluations, career advancement, or other employment-related decisions made by peers or supervisors.

### Group Assignments and Study Medications

Participants were randomized by the Penn Investigational Drug Service (IDS) in a 1:1 ratio to receive either hydroxychloroquine, 600 mg, daily, or placebo in blocks of 8 using established randomization software (SealedEnvelope.com; Clerkenwell Workshops). The IDS staff kept the randomization assignments concealed from study staff and investigators until interim analyses. Participants remained masked until study completion. Participants assigned to the hydroxychloroquine arm received hydroxychloroquine 200-mg tablets (provided by Sandoz, a division of Novartis Pharmaceuticals), with instructions to take 3 tablets once a day with food. Participants assigned to the placebo arm received custom-molded identically sized and shaped microcrystalline cellulose tablets (prepared for this trial by Temple IDS; Temple University; Philadelphia, Pennsylvania) and given identical instructions.

### Testing and Follow-up Procedures

At the time of randomization (baseline), 4 weeks, and 8 weeks, participants underwent study-specific NP swab testing for SARS-CoV-2 via a Clinical Laboratory Improvement Amendments–approved RT-PCR test (Quest Diagnostics). Study-specific NP swabs were obtained by trained members of the investigative staff using standard flocked tapered swabs (Quest Diagnostics) and placed immediately in a viral transport medium on ice for testing. Participants who developed COVID-19 symptoms were referred to Penn’s occupational medicine department for an urgent NP swab independent of the scheduled study procedures. At baseline, 4 weeks, and 8 weeks, serologic testing for 3 antibodies was performed: anti–nucleocapsid IgG, anti–spike protein receptor-binding domain (RBD) IgM, and anti-RBD IgG. Electrocardiographic (ECG) assessments were initially not required for enrollment according to the guidelines of the American College of Rheumatology for the use of hydroxychloroquine in an ambulatory population. During study conduct, other reports raised concerns for possible QT interval prolongation with use of hydroxychloroquine^16^; thus, the protocol was amended, and we instituted a 6-lead ECG evaluation at baseline and 4-week follow-up for participants using a Bluetooth ECG recorder (AliveCor). Electrocardiogram results were reviewed by a masked cardiologist study investigator (M.H.) to quantify corrected QT intervals (QTc) using an electronic caliper system (EP Calipers, version 2.0; EP Studios Inc).

All enrolled participants were contacted weekly by study coordinators to review daily pill diaries and adverse event standardized questionnaires. Any potential adverse events reported to the coordinators were relayed to study investigators, who then called participants to confirm and document adverse effects and determine the grade according to the Common Toxicity Criteria for Adverse Events (version 5.0)^17^ and the probability of attribution to study treatment. The highest grade of an adverse event that was experienced by each participant and deemed possibly related to the study drug was reported.

### Outcome Measures

The primary outcome was the rate of conversion to SARS-CoV-2–positive status via NP swab in enrolled participants during the 8 weeks of study participation. Participants were evaluable for the primary outcome if they had a negative result for the SARS-CoV-2 PCR test at baseline, took at least 1 dose of study medication, and had the opportunity to complete 8 weeks of the study. Secondary outcomes included the adverse event rate, rate of serologic antibody positivity for either nucleocapsid or spike protein antigens, ECG changes after 4 weeks of treatment, and clinical outcomes for any participants who became SARS-CoV-2 positive and/or developed COVID-19 symptoms within the 8-week study period.

### Statistical Power and Analysis

With the assumption of a 10% infection rate in the HCW population, we considered rejecting the null hypothesis if the infection rate was 1% with hydroxychloroquine treatment. With a planned enrollment of 200 participants (hydroxychloroquine arm and placebo arm, each with 100 participants), a 1-sided *z* test (α = .05) comparing the infection rates in the 2 groups would have an 80% power to detect a significant difference when the difference in the population rates was at least 9%. The study protocol followed a group sequential design that allowed for 2 interim analyses (after 50 and 100 participants enrolled, respectively) and permitted findings of early efficacy or futility before trial completion. Control of error rates was accomplished through the regulated spending of portions of α and β at each interim analysis and the final analysis (with a total of 5% and 20% across the trial, respectively). That pattern of spending was determined a priori during trial planning; 1-sided cutoff levels for *z* scores in each direction were established to determine early significance or futility. An early futility decision indicates that the infection rates diverge sufficiently from the original assumptions such that it would be impossible or extremely unlikely to detect a statistically robust difference were the trial to continue. For the second interim analysis, the *z* score needed to achieve early success was high (2.58), corresponding to a *P* value of .005. The *z* score needed to declare futility was a more modest value of opposite polarity (−0.27). If the *z* score were lower (more negative) than −0.27, futility would be determined, as it would be very unlikely to achieve significance under the original assumptions. At each interim analysis, the *z* score and all other safety and efficacy data were reported by the study team to the DSMB, which would recommend continuing or halting the study. Statistical analyses were conducted using Stata, version 16.1 (StataCorp).

## Results

**Figure 1.**
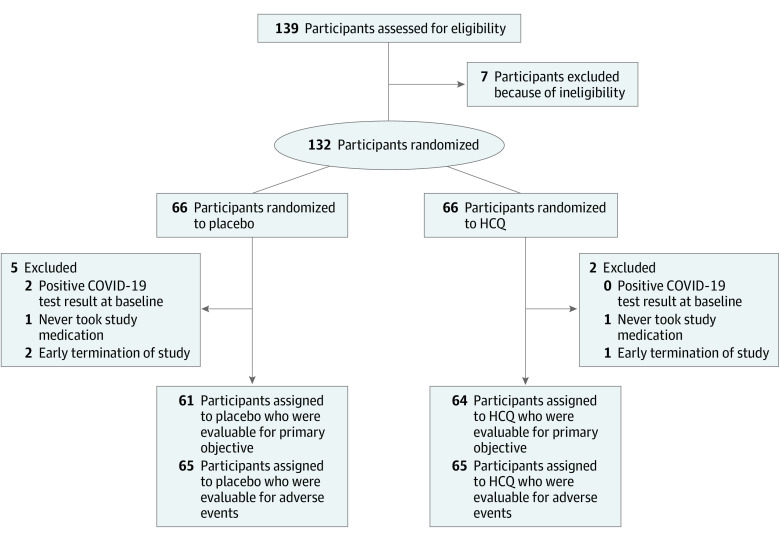
Participant Enrollment and Treatment Assignment COVID-19 indicates coronavirus disease 2019; HCQ, hydroxychloroquine.

Between April 9, 2020, and July 14, 2020, 139 participants provided consent (Table 1); the last follow-up assessment was completed August 4, 2020. During this study period, the Philadelphia region, including Penn Medicine hospitals, experienced a peak of infection rates followed by a decline in cases (eFigure 1 in Supplement 3). Study accrual mirrored the peak and decline of infection rate in the area (eFigure 1 and eFigure 2 in Supplement 3). Of the 139 patients who provided consent, 7 (5.0%) did not meet eligibility criteria; therefore, 132 participants were randomized. Five participants assigned to receive placebo (including 2 participants with positive test results for SARS-CoV-2 by RT-PCR at baseline) and 2 participants assigned to receive hydroxychloroquine were not evaluable for the primary outcome (Figure 1). Thus, 64 participants in the hydroxychloroquine treatment arm and 61 participants in the placebo arm were evaluable for the primary outcome (n = 125). The median age of the study population was 33 years (range, 20-66 years). The HCWs enrolled were predominantly women (91 [69%]), White (109 [83%]), and without preexisting medical problems (94 [71%]). Most participants worked as nurses or physicians in the emergency department (74 [56%]) or on internal medicine wards dedicated to treating patients with COVID-19 (35 [37%]).

**Table 1.** Baseline Characteristics of Randomized Participants

Characteristic	All participants (n = 132)		HCQ (n = 66)		Placebo (n = 66)		*P* Value
	No.	No. (%)	No.	No. (%)	No.	No. (%)	
Age, median (range), y	132	33 (20-66)	66	31 (20-66)	66	34 (23-62)	.13
Weight, median (range), kg	117	75 (50-190)	54	75 (53-190)	63	75 (50-145)	.36
BMI, median (range)	116	25 (19-50)	54	26 (19-37)	62	26 (20-50)	.30
Women	132	91 (69)	66	54 (82)	66	37 (56)	.001
Current smoker	132	0	66	0	66	0	NA
Coexisting conditions							
Asthma	132	23 (17)	66	9 (14)	66	14 (21)	.26
Diabetes		4 (3)		1 (2)		3 (5)	.31
Hypertension		17 (21)		3 (5)		14 (21)	.004
None		94 (71)		54 (82)		40 (61)	.01
Practice location							
Emergency department	132	74 (56)	66	38 (58)	66	36 (55)	.98
Internal medicine ward		35 (37)		17 (26)		18 (27)	
Intensive care unit/anesthesia		12 (9)		6 (9)		6 (9)	
Labor and delivery		11 (8)		5 (7)		6 (9)	
Occupation							
Nurse	132	86 (66)	66	46 (70)	66	42 (64)	.61
Physician		27 (21)		11 (17)		16 (24)	
Certified nursing assistant		4 (3)		2 (3)		2 (3)	
ED technician		4 (3)		3 (4)		1 (2)	
Physician assistant		1 (1)		1 (2)		0	
Respiratory therapist		8 (6)		3 (4)		5 (7)	
Race							
White	132	109 (83)	66	55 (83)	66	54 (82)	.60
Asian		14 (11)		7 (11)		7 (11)	
Black or African American		4 (3)		3 (4)		1 (2)	
Latinx		2 (2)		0		2 (3)	
Mixed heritage		3 (2)		1(2)		2 (3)	

Abbreviations: BMI, body mass index (calculated as weight in kilograms divided by height in meters squared); ED, emergency department; HCQ, hydroxychloroquine; NA, not applicable.

Most of the 125 participants evaluable for the primary end point completed the study. However, 22 of 125 participants (17.6%) discontinued study treatment early (eTable 1 in Supplement 3), with similar discontinuation rates between the hydroxychloroquine (12 of 64 [19%]) and placebo (10 of 61 [16%]) treatment arms (*P* = .73). All participants who discontinued treatment were followed for the intended 8-week study period and either agreed to complete the study procedures or provide information about COVID-19 symptoms and additional testing performed outside of the study.

The conversion of participants to SARS-CoV-2 positive status was determined either by study-administered NP swabs conducted at 4 weeks and 8 weeks or, if the participant developed symptoms, referral to the occupational medicine department for a NP swab. The rate of COVID-19 positivity (Table 2) was similar in the hydroxychloroquine and placebo arms (6.3% vs 6.6%; *P* > .99), with infections occurring throughout the 8-week period. None of the 8 participants with COVID-19 required hospitalization; all were either asymptomatic or had mild disease and fully recovered (eTable 2 in Supplement 3).

**Table 2.** Primary Outcome of SARS-COV-2 Positivity During 8-Week Study Period^a^

Time	HCQ (n = 64)				Placebo (n = 61)				Total positive
	Total tested	RT-PCR		Withdrew	Total tested	RT-PCR		Withdrew	
		Positive	Negative			Positive	Negative		
Week 1	1	1	0	4	1	1	0	1	2
Week 2	0	0	0	1	0	0	0	4	0
Week 3	0	0	0	4	0	0	0	2	0
Week 4	54	1	53	3	52	0	52	2	1
Week 5	1	1	0	0	0	0	0	0	1
Week 6	2	1	1	0	0	0	0	1	1
Week 7	0	0	0	0	0	0	0	0	0
Week 8	51^b^	0	51^c^	0	54^d^	3	51^c^	0	3
Total, No. (%)	NA	4 (6.3)	NA	12 (19)	NA	4 (6.6)	NA	10 (16)	8 (6.4)
SARS-CoV-2 positive	NA								NA
Absolute difference, % (95% CI)									−0.3 (−8.9 to −8.3)
*P* value									>.99

Abbreviations: HCQ, hydroxychloroquine; NA, not applicable; RT-PCR, reverse-transcriptase polymerase chain reaction; SARS-CoV-2, severe acute respiratory syndrome coronavirus 2.

^a^ Participants were tested at weeks 4 and 8; participants evaluated at these periods represent study-mandated testing, whereas participants evaluated at other weeks represent testing at occupational health or other clinical sites because of presumed symptoms or exposure.

^b^ One participant in the HCQ arm completed the study but did not have RT-PCR evaluation.

^c^ In each arm, some participants who voluntarily withdrew returned for RT-PCR testing and all had negative results; all withdrawn participants were contacted at 8 weeks to confirm their lack of symptoms.

^d^ Two participants in the placebo arm who voluntarily withdrew before 4 weeks had testing at 8 weeks but not at 4 weeks.

We conducted 2 preplanned interim analyses to determine if early termination was warranted because of efficacy or futility (eFigure 3 in Supplement 3). At the second interim analysis, conducted after 100 participants had completed the 8-week study period, 4 participants assigned to hydroxychloroquine and 3 participants assigned to placebo had converted to positive SARS-CoV-2 status, yielding a *z* score of −0.42 (odds ratio, 0.72), below the lower boundary *z* = −0.27 for futility. After reviewing the findings of the second interim analysis, the DSMB recommended early termination of the study and that the most recently enrolled participants (n = 3) discontinue study procedures immediately; 32 participants near completion were allowed to finish study procedures.

Serological testing for the presence of anti–spike protein RBD IgM and IgG and nucleocapsid protein IgG (eTable 3 in Supplement 3) demonstrated that only 2 participants had anti–nucleocapsid IgG at baseline. Both participants had a negative SARS-CoV-2 RT-PCR test result, and these participants did not possess anti–spike protein RBD IgG at baseline. At the end of the 8 weeks, there were more positive participants treated with hydroxychloroquine (4 [7.4%]) compared with placebo (2 [3.7%]) who had an IgG antibody against SARS-CoV-2 (*P* = .40). All participants who developed antibodies also converted to SARS-CoV-2 positive status (eTable 4 in Supplement 3).

**Figure 2.**
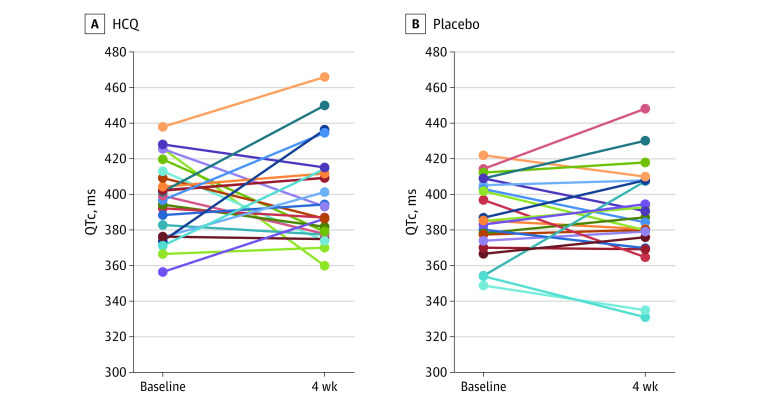
Corrected QT Interval (QTc) Assessment of Study Participants, Measured at Baseline and 1 Month The hydroxychloroquine (HCQ) and placebo arms both had 23 participants who received pretreatment and posttreament electrocardiograms. Each patient is represented with a different colored line.

At least 1 dose of study medication was taken by 65 participants in each arm; therefore, these participants were evaluable for adverse events (Table 3). The mean (SD) percentage of total pill counts prescribed that were actually taken during study treatment was 97% (8%) (hydroxychloroquine) and 98% (4%) (placebo). No participants in this study experienced grade 3 or 4 adverse events on the Common Toxicity Criteria for Adverse Events scale, hospitalizations, or death. However, there was a significant increase in any adverse events in the hydroxychloroquine arm vs placebo (45% vs 26%; *P* = .03), with increased diarrhea in participants receiving hydroxychloroquine compared with placebo (32% vs 12%; *P* = .01). No cardiac events (eg, syncope and arrhythmias) were observed. There was no significant difference in the median of changes in QTc between the hydroxychloroquine and placebo arms (4 milliseconds; 95% CI, −9 to 17; vs 3 milliseconds; 95% CI, −5 to 11; Wilcoxon 2-sample *t* test, *P* = .98; Figure 2).

**Table 3.** Adverse Events in Evaluable Participants

Characteristic	No. (%)						*P* value
	HCQ (n = 65)		Placebo (n = 65)		Any grade		
	Grade 1	Grade 2	Grade 1	Grade 2	HCQ	Placebo	
Percentage compliance with prescribed medication while on study, mean (SD)	NA				97 (8)	98 (4)	.37
Adverse event							
Abdominal pain	2 (3)	2 (3)	0	0	4 (6)	0	.12
Anorexia	7 (11)	0	2 (3)	0	7 (11)	2 (3)	.17
Chest pain	1 (2)	0	1 (2)	0	1 (2)	1 (2)	>.99
Constipation	0	0	1 (2)	0	0	1 (2)	>.99
Diarrhea	13 (20)	8 (12)	7 (11)	1 (2)	21 (32)	8 (12)	.01
Dizziness	1 (2)	0	0	0	1 (2)	0	>.99
Fatigue	2 (3)	0	0	0	2 (3)	0	.50
Gastroesophageal reflux	2 (3)	0	0	0	2 (3)	0	.50
Headache	0	0	2 (3)	2 (3)	0	4 (6)	.12
Nausea	6 (9)	0	5 (8)	0	6 (9)	5 (8)	.75
Paresthesia	1 (2)	0	0	0	1 (2)	0	>.99
Rash	2 (3)	1 (2)	1 (2)	0	3 (5)	1 (2)	.62
Throat tightness	0	0	0	1 (2)	0	1 (2)	>.99
Participants with any AE	NA				29 (45)	17 (26)	.03

Abbreviations: AE, adverse event; HCQ, hydroxychloroquine; NA, not applicable.

## Discussion

Among hospital-based HCWs at high risk of exposure to SARS-CoV-2, hydroxychloroquine, 600 mg, daily, for 8 weeks did not reduce the incidence of SARS-CoV-2 infection compared with placebo. Our findings are consistent with what is to our knowledge the only other randomized COVID-19 prophylaxis trial published to date.^14^ In that study, Boulware et al^14^ randomized 821 asymptomatic adults to hydroxychloroquine or placebo following a postexposure prophylaxis strategy in which participants self-identified as having a significant exposure and were treated with a 5-day course of hydroxychloroquine or placebo. The treatment protocol allowed for therapy initiation up to 4 days after exposure; more than 50% of participants started taking medication 3 to 4 days after exposure. This time variability prompted a critique^15^ that delayed initiation of hydroxychloroquine may have missed a key biologic window to prevent transmission. We elected to follow a pre-exposure prophylaxis strategy under the presumption that (1) prevention might depend on the timing of therapy, and (2) clear identification of a true exposure likely to result in transmission is challenging.

Our study also differed from the work of Boulware et al^14^ regarding SARS-CoV-2 testing. Following a pragmatic study design, there was a paucity of viral testing at either study initiation or at the time of the primary outcome (laboratory-confirmed transmission or illness compatible with COVID-19). Fewer than 25% of participants with a positive primary outcome had laboratory confirmation of SARS-CoV-2. Thus, participants may have become SARS-CoV-2 positive while remaining asymptomatic (contributing to type II error), or participants may have contracted another viral illness resulting in fever or cough that was not COVID-19 (contributing to type I error). By contrast, in our study, all participants had baseline SARS-CoV-2 testing and were excluded if found to have a positive result, and our primary outcome was defined as laboratory-confirmed SARS-CoV-2 transmission.

Similar to other studies of hydroxychloroquine for either viral prophylaxis or COVID-19 treatment, we found that the medication was generally well tolerated, with the exception that patients treated with hydroxychloroquine, 600 mg, for 8 weeks experienced significantly higher rates of grade 1 to 2 diarrhea than patients treated with placebo. In addition, we found no significant differences in cardiac adverse events between the hydroxychloroquine and placebo groups. Myocardial inflammation associated with SARS-CoV-2 infection may increase susceptibility to potential cardiac effects of hydroxychloroquine.^18^ Therefore, the lack of QTc prolongation or arrythmias in our study’s cohort cannot be used to infer cardiac safety of hydroxychloroquine for active COVID-19 treatment. Furthermore, some studies have involved the combined use of azithromycin, a known QTc-prolonging compound, and hydroxychloroquine^19^; azithromycin use was an exclusion criterion in our investigation.

Prophylaxis studies of infectious diseases are highly sensitive to disease frequency. In Pennsylvania, daily COVID-19 incidence fell during the course of enrollment (eFigure 1 in Supplement 3), starting at 14.8 cases per 100 000 population per day on April 9, 2020, and ending at 7.1 cases per 100 000 population per day on July 14, 2020.^20^ The overall SARS-CoV-2 infection rate in the study cohort was 6.4%; it is possible that a study of similar design conducted in a community with higher disease prevalence might yield a higher HCW infection rate and possibly more power to detect a prophylactic benefit from hydroxychloroquine. Alternatively, it is possible that uniform use of PPE and hand hygiene was sufficiently effective to reduce HCW infection to low levels, as seen in our study population.

### Limitations

Our study has important limitations. Our study was likely established with insufficient power. Given the small sample size, we cannot exclude the possibility of an undetected modest potential prophylactic effect of hydroxychloroquine. We did not attempt to quantify the frequency of participant exposure or specific timing of exposures. The cohort largely comprised young healthy HCWs and thus may not be generalizable to other populations with increased risk because of advanced age or additional comorbidities. Both study hospitals were located in Philadelphia and may not be representative of COVID-19 prevalence and exposure risk in other geographical areas. We cannot exclude the possibility that a lower or intermittent dose of hydroxychloroquine would be more effective at prevention, although a recent preclinical investigation in a COVID-19 macaque model did not find differences in antiviral activity with varied hydroxychloroquine dosing.^21^ Ongoing prophylaxis trials using hydroxychloroquine will be important to address these limitations.^22,23^

## Conclusions

This randomized clinical trial did not detect a reduction in SARS-CoV-2 transmission with prophylactic administration of hydroxychloroquine, and all participants who did contract SARS-CoV-2 were either asymptomatic or had mild disease courses with full recoveries. As such, we cannot recommend the routine use of hydroxychloroquine among HCWs to prevent COVID-19.

## Notes

Supplement 1. Trial Protocol

Supplement 2. Statistical Analysis Plan

Supplement 3. **eFigure 1.** Overall study design and schedule of study procedures **eFigure 2.** COVID-19 prevalence and accrual **eFigure 3.** Efficacy and futility bounds that guided early termination **eTable 1. **Treatment discontinuation in subjects evaluable for the primary outcome **eTable 2. **Subjects who were infected with SARS-CoV-2 **eTable 3. **Antibodies against SARS-CoV-2 nucleocapsid and spike proteins **eTable 4.** Correlation between PCR and antibody positivity in all subjects with either test positive

Supplement 4. Data Sharing Statement
