# Supplementary material for: Efficacy and Safety of Hydroxychloroquine vs Placebo for Pre-exposure SARS-CoV-2 Prophylaxis Among Health Care Workers: A Randomized Clinical Trial
Source: JAMA Intern Med. 2020 Sep 30;181(2):1–8. doi: 10.1001/jamainternmed.2020.6319 (PMC7527945; doi:10.1001/jamainternmed.2020.6319)
Supplement: Supplement 2. — Statistical Analysis Plan [file jamainternmed-e206319-s002.pdf]

Supplement 2 Abella et al.

**Sponsor: Ravi K. Amaravadi, MD**<br>**Institution: University of Pennsylvania**	
**Protocol Identifier:**	PATCH Trial NCT04329923
**File Type:**	Statistical Analysis Plan (SAP)

Title: **Hydroxychloroquine for Pre-exposure SARS-CoV-2 Prophylaxis among Healthcare Workers: A Randomized Clinical Trial**

A Sub-Study of the Prevention and Treatment of COVID-19 with Hydroxychloroquine (PATCH)

Description:

- The SAP aims at describing the analysis planned for protocol NCT04329923 and the information to be reported for the clinical study.
- The SAP will describe the analysis on safety, efficacy and related endpoints for the present study.

Introduction

This document is a statistical analysis plan (SAP) of NCT04329923 PATCH trial Sub-Study 3: a double-blind randomized controlled clinical trial of hydroxychloroquine for pre-exposure prophylaxis to prevent transmission of SARS-CoV-2. The SAP summarizes the study design and objectives and aims at providing a detailed description of the statistical analyses planned for the protocol.

Study Design

This study is a multi-hospital single-health system randomized, double blind, placebo-controlled, group-sequential trial that aims at studying the efficacy and safety of hydroxychloroquine (HCQ) 600 mg daily compared to inert placebo tablets with matched size/shape as a pre-exposure prophylaxis treatment for at-risk hospital-based adult health care workers (HCWs).

In the study, the investigators plan to recruit 200 HCWs who work full-time in the hospital setting, who will be randomly assigned to the trial group or the control group with a proportion of 1:1. The experimental group will be treated with HCQ and the control group will be treated with placebo. The subjects who pass the screening and meet the eligibility criteria will join the study and will receive study medication to take at home for eight weeks. All subjects will be contacted weekly by study staff to ensure compliance with study procedures and to monitor for adverse effects. At baseline, 4 weeks and 8 weeks, all subjects will undergo nasopharyngeal swab (NP) testing for SARS-CoV-2

Protocol amendments and statistical plan			
Protocol version	Date of Institutional Review Board Approval	Changes to sub-study 3	Changes to Statistical Plan
1	April 2, 2020		
2	April 21, 2020	Baseline, one month ECG	QTc change a new secondary endpoint
3	May 6, 2020	Expansion of eligibility criteria to health care workers beyond Emergency department and infectious disease ward	None
4	May 24, 2020	None	None
5	June 23, 2020	1.DSMB charter<br>2. Clarification that PCR+ positive patients at baseline are not evaluable for primary endpoint<br>3. secondary objective of # shifts missed is removed<br>4. clarification that subjects that discontinue study medication can still complete study procedures	1. Independent DSMB recommends early termination or continued accrual<br>2. Evaluable patients for primary outcome finalized

Study Objectives

PRIMARY OBJECTIVES

Rate of COVID-19 infection (cumulative incidence) during eight weeks of treatment or followup, as determined by NP swab testing performed by study staff at the 4-week and 8-week timepoints OR confirmed positive NP swab testing via a CLIA-approved clinical test performed before the 8-week timepoint.

SECONDARY OBJECTIVES

Rate of treatment related adverse events, as patient-reported outcomes obtained during weekly telephone calls to all subjects, following the Common Terminology Criteria for Adverse Events version 5.0 (CTCAE v 5.0).

CORRELATIVE OBJECTIVE

To bank serially collected samples to enable correlative science related this trial

Statistical Hypotheses

We hypothesize that there will be a statistically significant fewer new SARS-CoV-2 positive cases in the HCQ-treated cohort compared to the placebo-treated cohort during the 8 weeks of follow up.

Sample size and statistical treatment

The transmission of SARS-CoV2 from patient to hospital worker depends on many factors including specifics of standard care to prevent transmission, but especially on the number of patients seen at a given hospital or outpatient practice. Across China the reported hospital worker infection rate is 3.8%, but in Wuhan it is reported as 58% at the height if the epidemic. We will use a 10% cumulative incidence as the null hypothesis. In order for HCQ to be considered effective our alternative hypothesis will be a 1% transmission rate. With a 1:1 randomization for the HCQ to control arms we would require a total of 200 hospital worker subjects total. With the placebo group of 100 subjects and the HCQ arm of 100 subjects, a one-sided z-test (alpha=0.05) comparing the rates in the two groups would have 84% power to detect a significant difference when the difference in the population rates is at least 9%.The design if akin to a randomized phase II design and not a randomized phase III design.

The hypothesis will be tested using a one-sided test, with a z-score corresponds to the log of the odds-ratio for remaining uninfected between the two groups. We will obtain the estimate of the odds ratio, and 95% CI, from logistic regression.

Randomization process

The study is a randomized, double-blind controlled trial. Randomization will be conducted via the Penn Investigational Drug Service, following a block randomization strategy. Fixed blocks of 8 will be assigned using well-established proprietary software for clinical trials (Sealed Envelope, London UK). Randomization will be kept confidential at the IDS until the pre-specified interim and final analyses. If any SAEs occur or if subjects become SARS-C0V-2 positive, those subjects can be unblinded by written request of the PIs to the IDS team.

General

Statistical analyses will be performed using Stata v16, SAS ® software v9.4 or later, or R. All statistical analyses will mirror those of the treatment groups of Hydroxychloroquine (HCQ) and Placebo.

Without any other specification, all statistical tests will conducted with a two-sided 5% error. The primary hypothesis will also be tested with a 5% type-one error, but that error will be one sided, corresponding to the nature of the interim analyses. Small portions of the type 1 and type 2 error will be spent on interim

analyses, following Hwang-Shih-DeCani alpha and beta spending rules, leaving error rates at the final analysis slightly reduced.

Prior to analysis, data will be screened, cleaned, and examined for outliers using standard methods. Demographic and outcome variables will be summarized overall and by treatment. Continuous measurements will be described by means ± standard deviations or medians with minimum and maximum or the interquartile range (IQR). Binary outcomes will be described by frequencies and proportions (percentages).

The results of randomization will be examined by comparing treatment arms for demographic and other relevant variables, to test for incidental bias. We will use two-sample statistics (t-tests or Wilcoxon tests) or contingency table methods (chi-square or Fisher's exact tests) as appropriate.

Primary Analysis

The primary analysis will consist of a z-test, corresponding to the log of the odds-ratio for the proportion well in the HCQ group, versus the proportion well in the placebo group at the end of 8 weeks. The estimate will be made using either logistic regression or directly using contingency table methods. Tests will be one-sided, with type one error based on the alpha spending plan, with an overall type one error of 5%.

Interim Analysis

We will perform two interim analyses at 25% and 50% completion, testing for early efficacy or futility, using z-score boundaries that follow Hwang-Shih-DeCani alpha and beta spending rules (Z-value boundaries are shown in the table below).[1,2] Following those rules, we have 84% power using 100% of the sample, if our infection rate in placebo compared to hydroxychloroquine is 10% versus 1% scenario is true. Under those circumstances, we have a 6% chance of stopping for early efficacy at the first interim analysis. Under normal circumstances (10% vs 1%), we have a 27% chance of stopping for early efficacy at the second interim analysis. We also have an 80% chance of stopping early for efficacy at the second interim analysis if the low dose arm really has 23% infection instead of 10%. Results of the interim analysis will be presented to the Data Safety and Monitoring Board (DSMB) by the study team. The DSMB will provide a recommendation of continuing or terminating the study early.

Z-Value Boundaries

	Boundaries		Information
Stage	Efficacy	Futility	Proportion
1	2.9473	-1.2318	0.2500
2	2.5825	-0.2676	0.5000
3	1.6664	1.6664	1.0000

P-Value Boundaries

	Boundaries		Information
Stage	Efficacy	Futility	Proportion
1	0.00160	0.89099	0.2500
2	0.00491	0.60550	0.5000
3	0.04782	0.04782	1.0000

References
1- Hwang, I. K., Shih, W. J., and De Cani, J. S. (1990). Group sequential designs using a family of Type I error probability spending functions. Statistics in Medicine 9, 1439– 45.
2- Jennison, C. and Turnbull, B. W. (2000). Group Sequential Methods with Applications to Clinical Trials. Chapman and Hall/CRC, Boca Raton, FL.

Final analyses

Upon completion of the trial and the necessary data cleaning, the dataset will be locked for final analysis. This may occur at the final sample size of N=200, or earlier in the case of a decision to halt for futility. This final analysis will be conducted by study staff under supervision of the PIs with full involvement of two study statisticians. PIs and statisticians will review all results and p-values before analysis is determined complete and before manuscript submission.

Analysis Set	Definition	Outcomes
Randomized patients	Any patient randomized	Demographics
Evaluable for primary outcome	Randomized covid-19 negative at baseline and took at least one dose of study drug, and either completed all study procedures or	Primary outcome
Evaluable for Safety	Randomized and took one dose of study drug	Adverse events
Evaluable for drug-induced QTc prolongation	Randomized COVID-19 negative at baseline, took at least one dose of study medication and had a baseline and one month EKG	Change in QTc
Evaluable for antibody prevalence	Subjects who had blood drawn at specified timepoint	Antibody prevalence

Missing Data

Missing data values will not be imputed. Only observed values will be used for data analysis and presentation.

Subject Disposition

The summary statistics of the subjects will include the following information:
- The number and percentage of subjects who complete the trial and who drop out before trial completion according to the treatment groups.
- The number and percentage of subjects per analysis population according to the treatment groups.

Protocol Deviations

All protocol violations will be listed. The sponsor and/or its designee will review the protocol violations before the database is locked, and determine whether the involved subjects will be excluded from the per protocol set.

Demographic and Baseline Variables

The following demographic and baseline variables collected prior to the start of study medication will be summarized by treatment group.

Sex
Age
Profession (MD, nurse, respiratory technician, etc)
Location of work (ICU, ward, emergency department, etc)
Past medical history

Baseline testing will include NP swab testing for SARS-CoV-2, serologic testing for antibodies against SARS-CoV-2, and ECG testing (protocol amended DATE to include as a baseline test).

Adverse event reporting

The timely reporting of adverse events (including hospitalizations and deaths) is required by the Food and Drug Administration. The reporting of medication toxicities and other adverse events is part of the data reporting for this study. The sponsor-investigator is responsible for ensuring that all adverse events (AEs) and significant adverse events (SAEs) that are observed or reported during the study are collected.

Adverse Events

An adverse event (AE) is any symptom, sign, illness or experience that develops or worsens in severity during the course of the study that does not necessarily have a causal relationship with this treatment. Intercurrent illnesses or injuries should be regarded as adverse events. Abnormal results of diagnostic procedures are considered to be adverse events only if the abnormality:

• results in study withdrawal
• is associated with a serious adverse event
• is associated with clinical signs or symptoms
• leads to additional treatment or to further diagnostic tests is considered by the investigator to be of clinical significance

Adverse Event Reporting Period

The study period during which adverse events must be reported is defined as the period from the initiation of the first study treatment to the last administration of study treatment.

Post-study Adverse Event.

All unresolved adverse events should be followed by the clinical investigator until the events are resolved, the participant is lost to follow-up, or the adverse event is otherwise explained. At the last scheduled visit, the investigator should instruct each participant to report any subsequent event(s) that the participant, or the participant’s personal physician, believes might reasonably be related to participation in this study.

Abnormal Laboratory Values.

A clinical laboratory abnormality should be documented as an adverse event if the abnormality is grade 1 or more and a change from baseline.

Recording of Adverse Events.

At each contact with the participant, the appropriately delegated and credentialed study team member must seek information on adverse events by specific questioning and, as appropriate, by examination. Information on all adverse events should be recorded immediately in the source document, and also in the appropriate adverse event module of the case report form (CRF). All clearly related signs, symptoms, and

abnormal diagnostic procedures results should be recorded in the source document, though should be grouped under one diagnosis.

All adverse events occurring during the study period must be recorded. Adverse events will be measured and graded in accordance with the CTCAE v 5.0. The clinical course of each event should be followed until resolution, stabilization, or until it has been determined that the study treatment or participation is not the cause. Serious adverse events that are still ongoing at the end of the study period must be followed up to determine the final outcome. Any serious adverse event that occurs after the study period and is considered to be at least possibly related to the study treatment or study participation should be recorded and reported immediately.

Serious Adverse Events

Adverse events are classified as serious or non-serious.
A serious adverse event is any AE that is:
• fatal
• life-threatening
• requires or prolongs hospital stay
• results in persistent or significant disability or incapacity
• a congenital anomaly or birth defect
• Suspected transmission of an infectious agent (eg, pathogenic or nonpathogenic) via the study drug is an SAE.
• an important medical event

Important medical events are those that may not be immediately life threatening, but are clearly of major clinical significance. They may jeopardize the participant, and may require intervention to prevent one of the other serious outcomes noted above. For example, drug overdose or abuse, a seizure that did not result in in-patient hospitalization, or intensive treatment of bronchospasm in an emergency department would typically be considered serious. Theft, sale, or use of the study product by any person other than the participant will be reported as a medically important event.

DSMB Description

The Data and Safety Monitoring Board (DSMB) for the Prevention And Treatment of COVID-19 with Hydroxychloroquine (PATCH) trial has been selected by the Sponsor- Principal Investigator Dr. Ravi Amaravadi and acts in an advisory capacity to Dr. Amaravadi for the PATCH trial to monitor participant safety, data quality and evaluate the progress of the study.

The DSMB will consist of 5 members:
Dr. John Younger, expertise in Emergency medicine, Chair of DSMB
Dr. Keith Hamilton, expertise in infectious disease
Dr. Jeffrey Morris: expertise in biostatistics,
Dr. Sunita Nasta: expertise in clinical trials and therapeutics
Dr. Rajat Deo: expertise in clinical trials and cardiology

This DSMB will be independent of the Sponsor, regulatory agencies, IRBs/ECs, and investigators.

DSMB Membership

Requirements for Membership

- Each member declares any conflicts of interest related to the study. Members will notify Sponsor of any change in conflict.
- Each DSMB member maintains confidentiality of DSMB communications.

Remuneration

No remuneration will be provided

Roles and Responsibilities

The primary charge of the DSMB is to monitor the study for subject safety. Formal DSMB safety reviews occur as outlined in the charter. Additional review may be required. The DSMB may monitor effectiveness outcomes to determine relative risk/benefit, futility, or for early termination due to overwhelming effectiveness.

The primary safety endpoint as well as guidance for the conduct of analyses and guidelines/stopping rules are established in the protocol and are reviewed by the DSMB.

The DSMB may also review data related to study conduct. Data to be reviewed and listed in the DSMB reports may include: enrollment rates over time, time from last subject enrolled to date of report (indication of delay between treatment or follow-up and reporting), summary of protocol violations as specified in monitoring reports generated by the Office of Clinical Research (OCR), completeness of treatment and follow-up visit data, and follow-up duration for the population included in the report. Protocol deviations, SAE reports, and assistance in day to day medical decision making on study participants will be handled by the Medical Monitor Dr. Sunita Nasta who is also a member of the DSMB. The DSMB will in turn be able to review formal reports generated by the OCR monitor and the interim analysis reports generated by the Sponsor-Investigator and his team.

Conflicts of Interest

Members comply with the conflict of interest policies specified by University of Pennsylvania to ensure members do not have serious scientific, financial, personal, or other conflicts of interest related to the conduct, outcome, or impact of the study according to the guidelines specified below (e.g., engaged in any simultaneously occurring competitive trials in any role that could pose a conflict of interest for this study).

The DSMB follows 42 C.F.R. Part 50, Subpart F and Responsible Prospective Contractors 45 C.F.R. Part 94.

As determined by the Sponsor, conflicts of interest and/or potential conflicts of interest are mitigated to the greatest extent, consistent with assembling a highly competent DSMB.

DSMB Meetings

First Meeting: The purpose of the first meeting is to review, discuss, and make recommendations about the Charter and protocol. In addition, a chairperson is elected.

Subsequent Meetings: The DSMB meets and reviews unblinded deidentified data provided in the form of a report generated by the sponsor at the timepoints detailed below. The unblinded assignments of subjects may only be shared with the minimum number of study team members needed to generate the report for the DSMB review.

Additional meetings or conference calls are scheduled as needed.

Substudy 3 (HCW pre-exposure prophylaxis)

1. Substudy 3 Interim analysis1: after 50 out of a target 200 evaluable patients have completed the study and the report is available for review
2. Substudy 3 Interim analysis 2: after 100 out of a target 200 evaluable patients have completed the study and the report is available for review

This DSMB is coordinated by the Sponsor. DSMB meetings are held by teleconference. All three members of the DSMB must be present to hold a DSMB meeting. Critical decisions of the DSMB are made by unanimous vote whenever possible, or majority vote if this is not possible.

While the Medical Monitor will review deviations, unanticipated serious adverse events and safety data, she may consult the DSMB as needed.

Meeting Format

DSMB meetings are organized into open and closed sessions. Data reviewed by the DSMB is provided by the Sponsor.

Open Sessions

Data presented in the open session may include enrollment data, individual adverse event data, baseline characteristics, overall data accuracy and compliance data or issues, and other administrative data. The DSMB also considers data from other studies or external sources as made available during its deliberations.

During the open sessions, prepared summary reports and tables are reviewed and discussed by the DSMB.

Facilitator(s) (e.g., member of Sponsor and/or investigator team responsible for the report preparation) and observers from University research oversight comittees may attend the DSMB meeting Open Sessions as necessary in order to facilitate data presentation, follow- up reporting, and answer questions posed by the DSMB.

Closed Sessions

The closed session is restricted to the DSMB members. Data which may compromise the integrity of the study (e.g., comparative data) is analyzed and discussed only in the closed session. Details of closed session deliberations (e.g., minutes) are considered privileged and not subject to disclosure except as required by law.

Meetings conclude with a recommendation to continue, modify, pause, or terminate the study. In addition, recommendations for modification, pause, or termination may be endorsed for perceived safety concerns based on clinical judgment.

DSMB Reports

Minutes of the open session are recorded and finalized. Minutes are approved by the chairperson and maintained by the Sponsor.

The minutes of the closed session are recorded. They are stored in a secure location by the DSMB chair, separately from the minutes of the open session. Once finalized and approved by the DSMB chair, they will be retained until the trial is completed.

DSMB Communication of Recommendations

A formal report containing the recommendations of the study is sent to the Sponsor.
If the DSMB recommends continuation of the study, the minutes and report for continuation are shared with the Sponsor no later than 3 business days after the meeting.

If the DSMB recommends modifications, pause, or termination of the study, the minutes and report are shared with the Sponsor no later than 1 business days after the meeting.

In addition, any findings that are considered to be serious and potentially consequential that require immediate action are promptly shared with the Sponsor.

DSMB Closeout

This study may be terminated under a variety of circumstances including, but not limited to, termination for overwhelming effectiveness, futility, or safety issues per protocol, poor enrollment, or DSMB monitoring guidelines.

Confidentiality

All data provided to the DSMB and all deliberations of the DSMB are privileged and confidential. The DSMB agrees to use this information to accomplish the responsibilities of the DSMB and will not use it for other purposes without written consent from the Sponsor.
