# Supplementary material for: Efficacy and Safety of Hydroxychloroquine vs Placebo for Pre-exposure SARS-CoV-2 Prophylaxis Among Health Care Workers: A Randomized Clinical Trial
Source: JAMA Intern Med. 2020 Sep 30;181(2):1–8. doi: 10.1001/jamainternmed.2020.6319 (PMC7527945; doi:10.1001/jamainternmed.2020.6319)
Supplement: Supplement 3. — eFigure 1. Overall study design and schedule of study procedures eFigure 2. COVID-19 prevalence and accrual eFigure 3. Efficacy and futility bounds that guided early termination eTable 1. Treatment discontinuation in subjects evaluable for the primary outcome eTable 2. Subjects who were infected with SARS-CoV-2 eTable 3. Antibodies against SARS-CoV-2 nucleocapsid and spike proteins eTable 4. Correlation between PCR and antibody positivity in all subjects with either test positive [file jamainternmed-e206319-s003.pdf]

Supplemental Online Content

Abella BS, Jolkovsky EL, Biney BT, et al; PATCH Investigators. Efficacy and safety of hydroxychloroquine vs placebo for pre-exposure SARS-CoV-2 prophylaxis among health care workers: a randomized clinical trial. *JAMA Intern Med*. Published online September 30, 2020. doi:10.1001/jamainternmed.2020.6319

eFigure 1. Overall study design and schedule of study procedures

eFigure 2. COVID-19 prevalence and accrual

eFigure 3. Efficacy and futility bounds that guided early termination

eTable 1. Treatment discontinuation in subjects evaluable for the primary outcome

eTable 2. Subjects who were infected with SARS-CoV-2

eTable 3. Antibodies against SARS-CoV-2 nucleocapsid and spike proteins

eTable 4. Correlation between PCR and antibody positivity in all subjects with either test positive

This supplemental material has been provided by the authors to give readers additional information about their work.

© 2020 Abella et al. *JAMA Internal Medicine.*

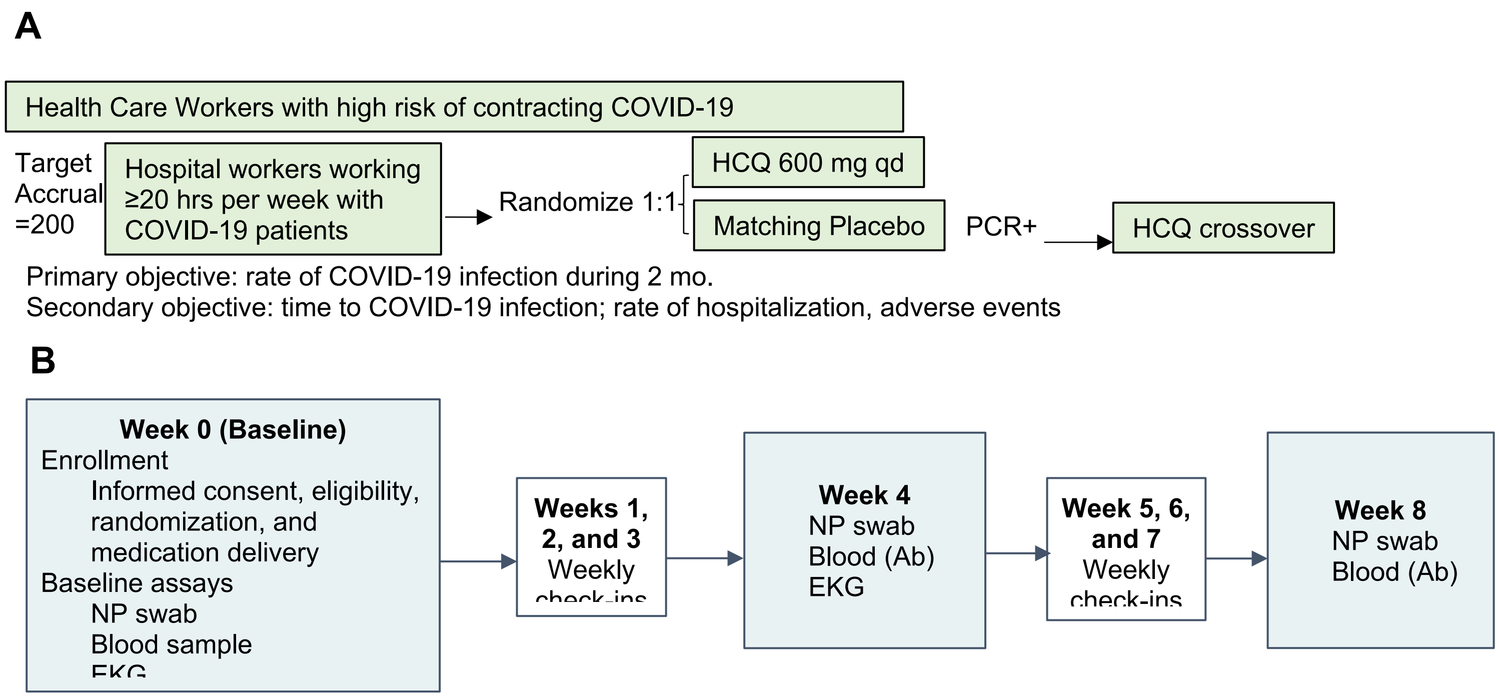

Supplemental eFigure 1. A. Overall Study Design **B.** Schedule of Study Procedures

© 2020 Abella et al. *JAMA Internal Medicine.*

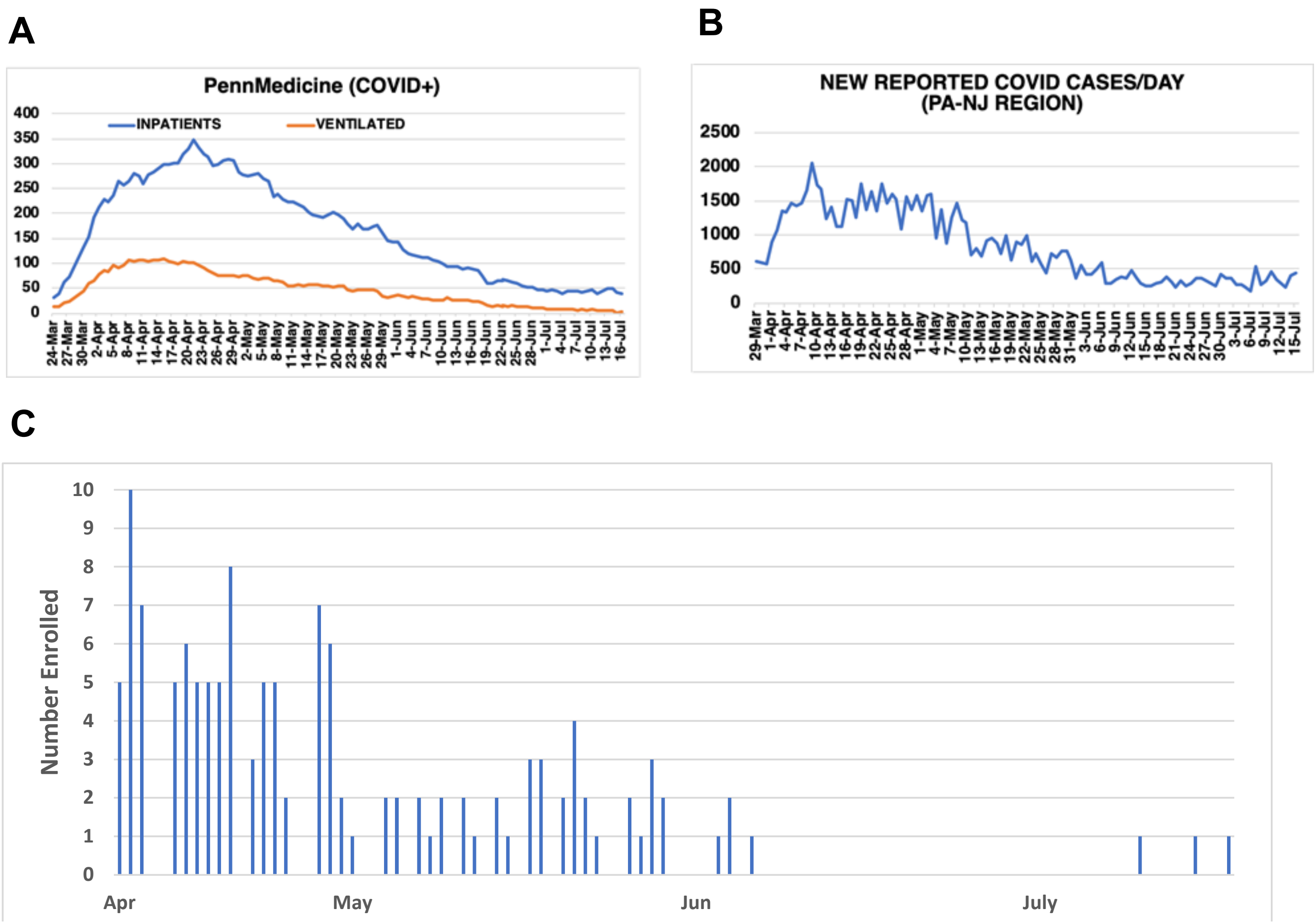

Supplemental eFigure 2. COVID-19 prevalence and accrual A. COVID-19 positive patients admitted to PennMedicine Hospitals from March to July 2020 B. Daily COVID-19 positive cases in the Pennsylvania and New Jersey Region surrounding University of Pennsylvania from March to July 2020. C. Enrollment by date of University of Pennsylvania health care workers to the PATCH study.

© 2020 Abella et al. *JAMA Internal Medicine.*

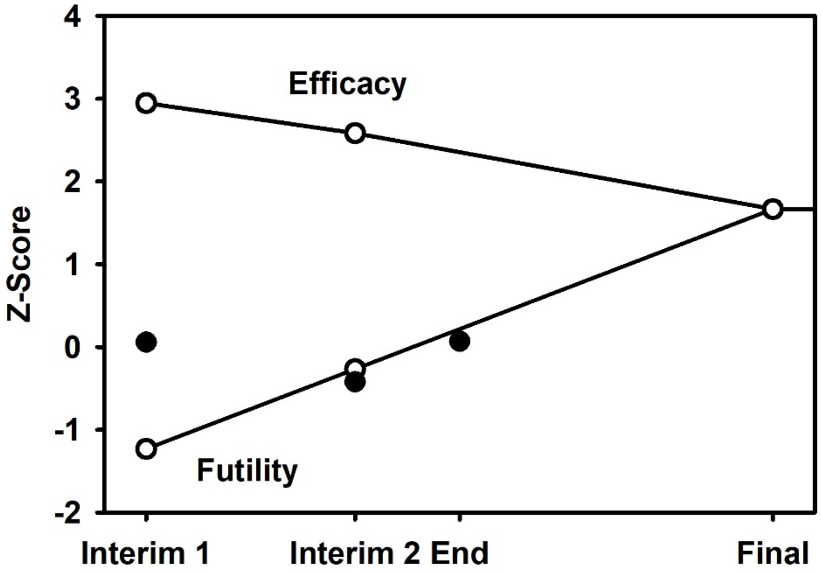

# COVID-19 +	Interim 1	Interim 2	End
HCQ	1	4	4
Placebo	1	3	4
Total N	50	100	125

Supplemental eFigure 3. Efficacy and futility bounds that guided early termination

© 2020 Abella et al. *JAMA Internal Medicine.*

eTable 1. Treatment discontinuation in subjects evaluable for the primary outcome						
Reason for discontinuation of assigned treatment	**HCQ (n=64)**	**Placebo (n=61)**	**Total**	**Completed study procedures**	**Did not complete study procedures**	**Did not complete study procedures and developed COVID-19 symptoms after treatment stopped**
Diarrhea	3 (20%)	1 (9%)	4 (18%)	1	3	1
Headache	0	2 (18%)	2 (9%)	1	1	0
Lack of compliance	1 (7%)	4 (36%)	5 (23%)	2	3	1
Personal reasons	8 (53%)	3 (27%)	11 (50%)	3	8	0
Total	12	10	22	7	15	2
Discontinuation rate	19%	16%				

© 2020 Abella et al. *JAMA Internal Medicine.*

eTable 2. Subjects who were infected with SARS CoV-2										
**Patient**	**Symptoms**	**PCR+**	**Anti-body+**	**Age**	**Sex**	**Job**	**Dept.**	**HCQ Crossover**	**Hospit-alized**	**Treatment**
Placebo 1	None	Wk 8	Wk 8	48	F	Physician	EM	Declined	No	None
Placebo 2	None	Wk 8	None	34	M	Nurse	IMW	Declined	No	None
Placebo 3	Nasal congestion, sore throat	Wk 8	None	34	F	Nurse	IMW	Declined	No	None
Placebo 4	Headache, body aches, fever, diarrhea, chills, SOB, fatigue, tachycardia, loss of taste and smell, myalgias, dizziness, pneumonia, chest pain, palpitations, abdominal pain	Wk 1	NA	54	F	Respiratory Therapist	ED	Declined	No	Joined Treatment Arm of PATCH (substudy1) randomized to placebo, progressive symptoms improved with HCQ crossover
HCQ 1	Cough, fatigue, headache, loss of taste and smell, sore throat	Wk 5	Wk 8	48	M	Physician	ED	N/A	No	None
HCQ 2	Fatigue, body aches, palpitations, DOE	Wk 4	Wk 4	27	F	Nurse	ED	N/A	No	None
HCQ 3	Fever, sore throat	Wk 6	Wk 8	35	F	Nurse	ED	N/A	No	None
HCQ 4	Back ache, fatigue, cough, fever, rigor, SOB, tachycardia, loss of appetite, pneumonia	Wk 1	NA	25	F	Nurse	IMW	N/A	No	Doxycycline

© 2020 Abella et al. *JAMA Internal Medicine.*

eTable 3. Antibodies against SARS-CoV-2 nucleocapsid and spike proteins

	HCQ (n=49)			Placebo (n=49)		
	**anti-N IgG**	**anti-RBD IgG**	**anti-RBD IgM**	**anti-N IgG**	**anti-RBD IgG**	**anti-RB IgM**
Baseline, number tested	66	66	66	66	66	66
Positive, n (%)	1 (1.5%)	0	0	1 (1.5%)	0	0
4 wk timepoint, number tested	54	54	54	53	53	53
Positive, n (%)	2 (3.7%)	1 (1.8%)	1 (1.8%)	1 (1.9%)	0	0
8 wk timepoint, number tested	54	55	55	54	54	54
Positive n (%)	4 (7.4%)	3 (5.4%)	2 (3.6%)	2 (3.7%)	1 (1.9%)	1 (1.9%)

RBD = Receptor-binding domain (spike protein)

N = Nucleocapsid protein6

© 2020 Abella et al. *JAMA Internal Medicine.*

eTable 4. Correlation between PCR and antibody positivity in all subjects with either test positive																			
		**SARS-CoV-2 RT-PCR Test**									**SARS-CoV-2 Antibody Test**								
											Baseline			4 Week			8 Week		
		**Timepoint (Week)**									α-N	α-RBD		α-N	α-RBD		αi-N	α-RBD	
**Treatment**	**Subject**	Baseline	1	2	3	4	5	6	7	8	IgG	IgG	IgM	IgG	IgG	IgM	IgG	IgG	IgM
**Placebo**	1A	P	-	-	-	-	-	-	-	-	N	N	N	-	-	-	-	-	-
	1B	P	-	-	-	-	-	-	-	-	N	N	N	-	-	-	-	-	-
	1C	N	-	-	-	N	-	-	-	N	P	N	N	P	N	N	P	N	N
	1D	N	-	-	-	N	-	-	-	P	N	N	N	N	N	N	P	P	P
	1E	N	-	-	-	N	-	-	-	P	N	N	N	N	N	N	N	N	N
	1F	N	-	-	-	-	-	-	-	P	N	N	N	-	-	-	N	N	N
	1G	N	P	-	-	-	-	-	-	-	N	N	N	-	-	-	-	-	-
**HCQ**	2A	N	-	-	-	N	P	-	-	N	N	N	N	N	N	N	P	P	P
	2B	N	-	-	-	N	-	-	-	N	P	N	N	P	N	N	P	N	N
	2C	N	-	-	-	P	-	-	-	N	N	N	N	P	P	P	P	P	N
	2D	N	-	-	-	N	-	P	-	P	N	N	N	N	N	N	P	P	P
	2E	N	P	-	-	-	-	-	-	-	N	N	N	-	-	-	-	-	-
P: Positive N: Negative																			

© 2020 Abella et al. *JAMA Internal Medicine.*
