# Supplementary material for: Efficacy and Safety of Hydroxychloroquine vs Placebo for Pre-exposure SARS-CoV-2 Prophylaxis Among Health Care Workers: A Randomized Clinical Trial
Source: JAMA Intern Med. 2020 Sep 30;181(2):1–8. doi: 10.1001/jamainternmed.2020.6319 (PMC7527945; doi:10.1001/jamainternmed.2020.6319)
Supplement: Supplement 4. — Data Sharing Statement [file jamainternmed-e206319-s004.pdf]

Data Sharing Statement

Abella. Efficacy and Safety of Hydroxychloroquine vs Placebo for Pre-exposure SARS-CoV-2 Prophylaxis Among Health Care Workers. *JAMA Intern Med*. Published September 30, 2020. 10.1001/jamainternmed.2020.6319

Data
Data available: Yes
Data types: Deidentified participant data
How to access data: https://clinicalresearch.itmat.upenn.edu/clinicaltrial/6407/covid19-a-trial-in-quarantined-subjects-with-covid19/
When available: With publication

Supporting Documents
Document types: Informed consent form
How to access documents: https://clinicalresearch.itmat.upenn.edu/clinicaltrial/6407/covid19-a-trial-in-quarantined-subjects-with-covid19/
When available: With publication

Additional Information
Who can access the data: researchers whose proposed use of the data has been approved
Types of analyses: for any purpose
Mechanisms of data availability: after approval of a proposal
